# Impact of Superabsorbent Polymers and Variety on Yield, Quality and Physiological Parameters of the Sugar Beet (*Beta vulgaris* prov. *Altissima* Doell)

**DOI:** 10.3390/plants10040757

**Published:** 2021-04-13

**Authors:** Vladimír Pačuta, Marek Rašovský, Beata Michalska-Klimczak, Zdzislaw Wyszyňski

**Affiliations:** 1 Department of Crop Production and Grassland Ecosystems, Faculty of Agrobiology and Food Resources, Slovak University of Agriculture in Nitra, Tr. A. Hlinku 2, 949 76 Nitra, Slovakia; vladimir.pacuta@uniag.sk; 2 Department of Agronomy, Faculty of Agriculture and Biology, Warsaw University of Life Sciences—SGGW, Nowoursynowska 166 St., 02-787 Warsaw, Poland; beata_michalska_klimczak@sggw.edu.pl (B.M.-K.); zdzislaw_wyszynski@sggw.edu.pl (Z.W.)

**Keywords:** sugar beet, superabsorbent polymers, LAI, PRI, yield, quality

## Abstract

In this study, we focus on the mitigation of the negative impact of drought using the application of superabsorbent polymers (SAPs) to seed. One way to monitor drought and quantify its impact on crops in field conditions is the nondestructive measurement of physiological processes of the crops using spectral indexes LAI and PRI during vegetation. Therefore, during 2018 and 2019, the increase in biomass and intensity of photosynthetic activity was monitored, and the effect of the SAPs application on the yield parameters of the sugar beet was evaluated in the trial conditions (control, SAPs) at the end of the vegetation period. Through statistical analysis, the significant impact (α ≤ 0.01) of SAPs application on the values of spectral indexes LAI and PRI as well as root and white sugar yields was found. Although the sugar content difference between SAPs and control conditions was not statistically significant, SAPs had a positive influence on the value of this parameter. It was found through periodic monitoring of spectral indexes during the growing period that the crop in the SAPs condition showed higher values of PRI at the beginning of vegetation, which was caused by the accumulation of moisture in the vicinity of the seed and subsequent faster growth of roots and photosynthetic apparatus. Moreover, the values of LAI were significantly higher (α ≤ 0.01) in the SAPs condition throughout the vegetation period. In the interaction evaluation, we confirmed that in both years the values of LAI were higher in the condition with SAPs compared with the control. In contrast, the PRI values were significantly different across conditions. The interaction of conditions with variety showed that the variety Brian obtained higher values of LAI and PRI in the SAPs condition. The correlation analysis found a positive correlation between spectral indexes LAI:PRI (*r* = 0.6184**), and between LAI:RY (*r* = 0.6715**), LAI:WSY (*r* = 0.5760**), and PRI:RY (*r* = 0.5038*), which confirms the close relationship between physiological processes in the plant and the size of its yield.

## 1. Introduction

From a global perspective, sugar beet (*Beta vulgaris* prov. *Altissima* Doel.) is considered one of the most important sugar-producing crops [1,2,3]. Furthermore, the influence of this crop is significant from the perspective of sustainability of field crop production systems because, in addition to producing sugar, it has potential as a source of biofuel [2]. From an economic perspective, the most important parameters are the root yield and sugar content [4]. Cultivation of sugar beets is widespread globally as sugar beets adapt to various climatic conditions [5] and are successfully grown in a wide variety of soil types [6]. In 2018, this crop was grown on 4.8 million hectares with EU28 accounting for approximately 36% [7].

The production potential of the sugar beet results from its ability to capture light radiation for photosynthesis, that is, the ability to close its canopy as fast as possible for the longest period possible, and thus optimize its intake of photosynthetic radiation [8]. An important parameter used to monitor the vegetation structure and growth of the sugar beet is the leaf area index (LAI) [9], which describes the leaf area of the crop per unit of the ground surface area [10]. To evaluate the light efficiency, the photochemical reflectance index (PRI), which uses the changes in reflectance in the interval 531 nm to 570 nm, can be used [11]. The PRI can function as an indicator of water stress in the early growth phases, but its use cannot be independent of light conditions [12]. When stress is induced, changing leaf and pigment structures, reflectance changes leading to changes in PRI [13]. Several studies show that the photochemical reflectance index is influenced by vegetation, structure [14,15] and by growth variables such as phenological phases and LAI [16]. To attain the potential of the sugar beet, all necessary growth factors must be available, and the crop must be able to use these factors. From this perspective, the concurrence of factors, such as the maximum use of sunlight, high water demand, and complete canopy closure, may be problematic [17].

The advancing climate change significantly influences the production process of field crops, and the sugar beet has not been spared, especially in central and southern parts of Europe [18]. One of the most important limiting factors influencing the sugar-beet harvest is drought [19,20]. Moreover, because of climate change, the consequences of droughts may become increasingly severe [21]. A water deficit develops when the level of transpiration is higher than water absorption, magnified by high temperatures and salinity [22]. Subsequently, the water deficit in the sugar beet causes reduction of the water potential of the leaves and relative water content, which decreases the growth speed of leaves and the root [23]. Furthermore, the drought stress influences the accumulation of sucrose in storage organs [24]. In recent years, a significant effort has been expended to reduce the impact of drought stress on the yield and quality of crops [25].

One way to increase the qualitative and quantitative yield parameters is the use of superabsorbents in the pre-sowing seed treatment [26,27]. Superabsorbent polymers (SAPs), known also as hydrogels [28], can absorb water and thus increase the irrigation interval. Moreover, they can decrease stress symptoms from drought [29], especially in regions with significant long-term moisture deficit [30]. SAPs can quickly absorb 10, sometimes up to 1000, times their own weight in water [31], especially owing to the high number of hydrophilic groups and the three-dimensional network [32]. SAPs are used in a wide variety of applications in medicine, industry, etc. [33]. In agriculture, the applications of SAPs have much potential as they are used as water-retaining materials and can hold a large amount of water and nutrients in an interaction with soil [34]. Subsequently, using controlled release, they provide these substances to the environment [35]. The most common polymers in agriculture are polyacrylamide and polyacrylate polymers [36]. The importance of SAPs as a soil conditioner has been investigated recently in drought areas during soil restoration [37] because it can effectively increase the soil moisture and porousness and improve the soil structure and nutrient efficiency [38]. The coating of seeds with SAPs increases their viability and provides water supply to the seeds, which is available during the germination phase [39]. In addition, the application of SAPs has a significant effect on the values of the leaf area index and other physiological parameters [40,41]. The application of SAPs in combination with fertilizers, which has improved the complex nutrition of the crops and reduced the water loss in the process of evapotranspiration, has been successful in practice [42]. Several studies have investigated the impact of SAPs on the growth of cultivated crops, such as common bean [43], common wheat [44,45], alfalfa [46], maize [37,47], sunflower [45], and onion [48].

Therefore, the main objective of this study was to determine and evaluate the impact of SAPs on the yield and physiological parameters of the sugar beet (root yield, sugar content, white sugar yield, leaf area index, and photochemical reflectance index) and to assess the mutual relationships of these parameters. All interventions and measurements were conducted in field conditions, which increases the value and applicability of all results in practice.

## 2. Results

**Figure 1:**
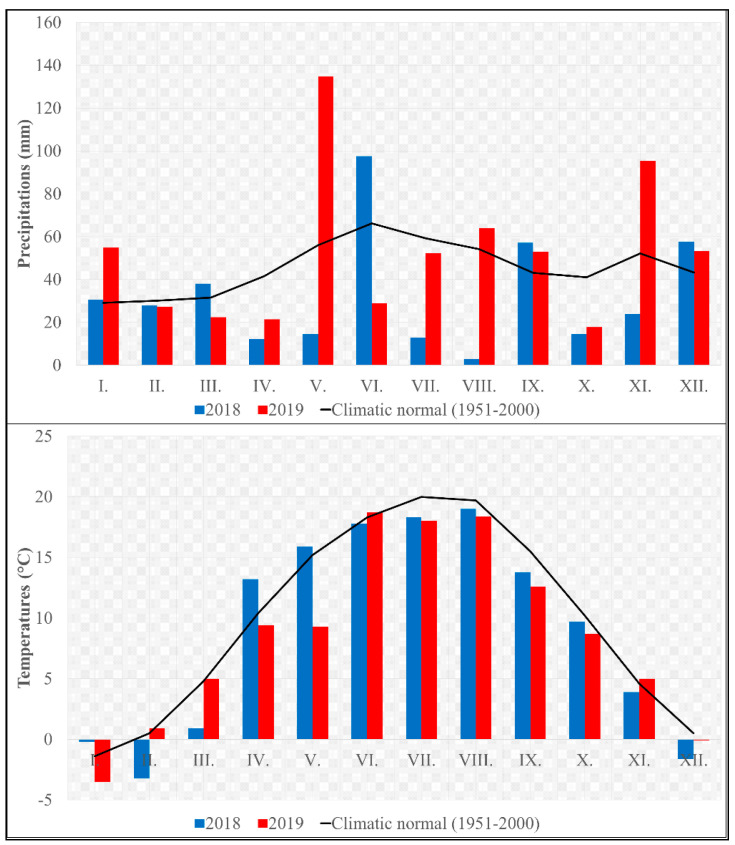
Precipitation (mm) and temperature (°C) variabilities of research years on experimental area. Included are long-term climatic normal values (1951 to 2000) of region. I.–XII. represent months.

To interpret the impact of drought on the quality and quantity of the yield, it is necessary to define the weather conditions of the observed years. Significant factors that influence the growth process of the sugar beet are temperature, rainfall, and soil moisture [49]. The temperature and rainfall were measured throughout 2018 and 2019 in the experimental area (Figure 1). However, the most important data are from the vegetation period (April to September). The total rainfall and sum of temperatures were considerably different between the observed years. In the vegetation year 2018, the recorded sum of temperatures was ∑ T_veg_ 2722.6 °C and the total rainfall was ∑ P_veg_ 197.4 mm, which was 86.1 °C more and 156.8 mm less, respectively, compared with the year 2019.

In particular, the distribution of rainfall in the experimental years significantly influenced the production of biomass, final yield, and production quality. The recorded values of rainfall and temperatures were compared with the climatic normal of the cultivation area from the years 1951 to 2000 [50]. The rainfall recorded at the beginning of the vegetation period (April to May) and in July 2018 was strongly subnormal. Furthermore, August 2018 can be described as an extremely dry month with exceptionally subnormal rainfall. In contrast, in the growing year 2019, normal rainfall was recorded in July to September compared with climatic normal; in May, above-normal rainfall was recorded, although the beginning of the vegetation (April) was marked by subnormal rainfall (Figure 1).

Different values were recorded in average monthly temperatures of the monitored years. Compared with the climatic normal, it can be stated that May and June 2018 were thermally normal, while August and September were subnormal, with a significant subnormal temperature profile in July. From the perspective of weather impact on plant development, the values from April 2018, when temperatures considerably above normal were recorded, are important. It can be assumed that this, in combination with markedly subnormal rainfall in the given year, had a significant impact on slowing the growth of sugar beets. In the growing year 2019, no temperatures above the long-term normal were recorded (Figure 1).

### 2.1. Leaf Area Index (LAI)

For many crops, LAI is one of the conclusive physiological indicators that demonstrate the intensity of biomass growth during the vegetation period. Furthermore, it is closely related with the processes of photosynthesis and respiration [51]. From the perspective of growing sugar beets, it is necessary for the canopy to reach the LAI value of 5 to 6 m^2^ m^−2^ as soon as possible and to maintain it for as long as possible. The limiting factor in dry and warm areas, which often negatively influences the optimum process of LAI, is the scarcity of rainfall combined with high temperatures during the summer months.

**Figure 2:**
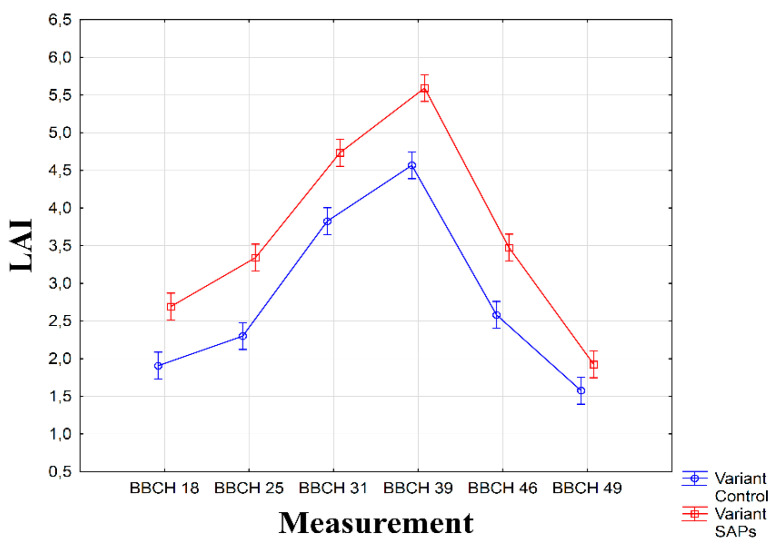
Interaction between experimental treatments and measurements for leaf area index (LAI). Figure was obtained at level of significance α = 0.01.

The statistical evaluation confirmed significant (α ≤ 0.01) impact of SAPs on LAI values (Table 1). The curve peak was recorded during the fourth measurement in both conditions, when the sugar beets were in the BBCH 39 phase. Significant differences (α ≤ 0.01) of values during individual measurements were found between the experimental conditions (Figure 2). Before and after the beginning of the experiments of both years, long periods of low rainfall were recorded and although in 2019 the last 10 days of May were favorable in terms of precipitation (Figure 1), the sugar-beet plants suffered drought stress, especially during critical growth phases. As can be seen in Figure 2, even in the early growth phases, the values of LAI were significantly higher in the conditions treated with SAPs compared with the control condition (α ≤ 0.01). This can be attributed to SAPs, which accumulated the soil moisture from the environment to the proximity of the seeds and roots of the sugar beets; these could then manage the drought stress significantly better than the plants in the control condition. The canopy in the condition treated with SAPs appeared in an earlier growth phase and attained significantly higher average values of LAI (α ≤ 0.01) throughout the entire vegetation period, which was demonstrated by the height of the crop at the end of the vegetation period.

**Figure 3:**
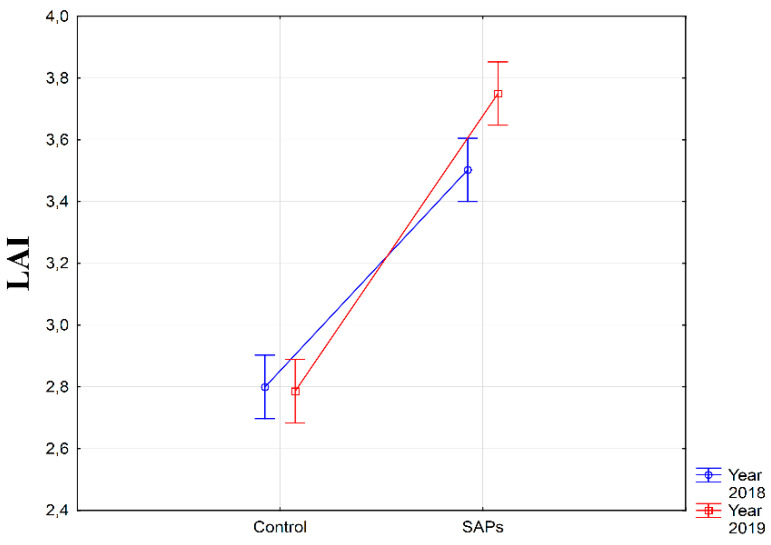
Interaction between experimental treatments and years for leaf area index (LAI). Figure was obtained at level of significance α = 0.01.

Monitoring of SAPs interactions with weather conditions during the experimental years confirmed significant differences (Figure 3) in the measured values of the index of leaf area. The highest average LAI value of 3.8 m^2^ m^−2^ from the perspective of year weather condition × SAPs interaction was found in 2019 on SAPs treatment with significant difference when compared with the other combinations (α ≤ 0.01). Thus, the influence of weather conditions of that year in combination with SAPs treatment significantly contributed to the values of this physiological parameter.

**Figure 4:**
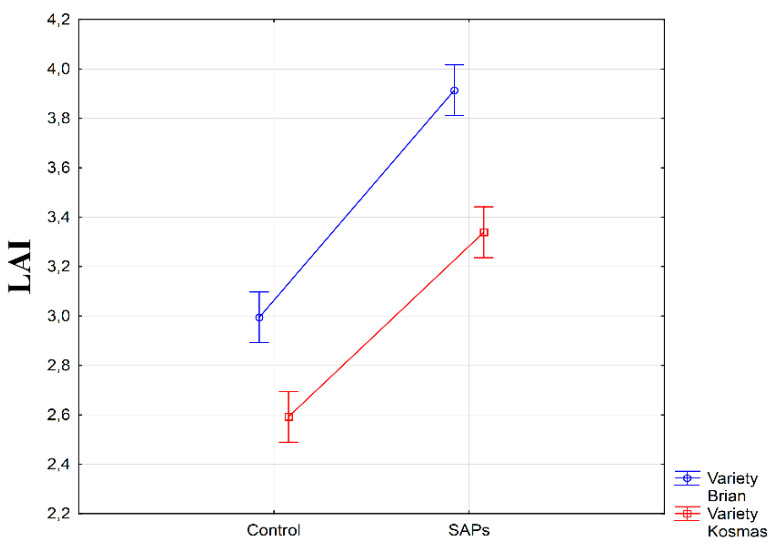
Interaction between experimental treatments and varieties for leaf area index (LAI). Figure was obtained at level of significance α = 0.01.

The SAPs application was investigated in two varieties of sugar beets. Both varieties, Kosmas and Brian, obtained significantly increased values of the LAI parameter (α ≤ 0.01) after the application of SAPs to the seed compared with the control condition (Figure 4). We found the highest average LAI value of 3.9 m^2^ m^−2^ in the interaction of variety Brian and SAPs treatment.

### 2.2. Photochemical Reflectance Index (PRI)

Although LAI is a reliable parameter to determine the canopy productivity, a further aim of this experiment was to determine changes in the internal physiological activity of the sugar beet, that is, the intensity of photosynthetic activity caused by SAPs application in dry and warm conditions. PRI is a reflectance indicator that can evaluate the level of intensity of photosynthesis using two near wavelengths (531 nm and 570 nm).

**Figure 5:**
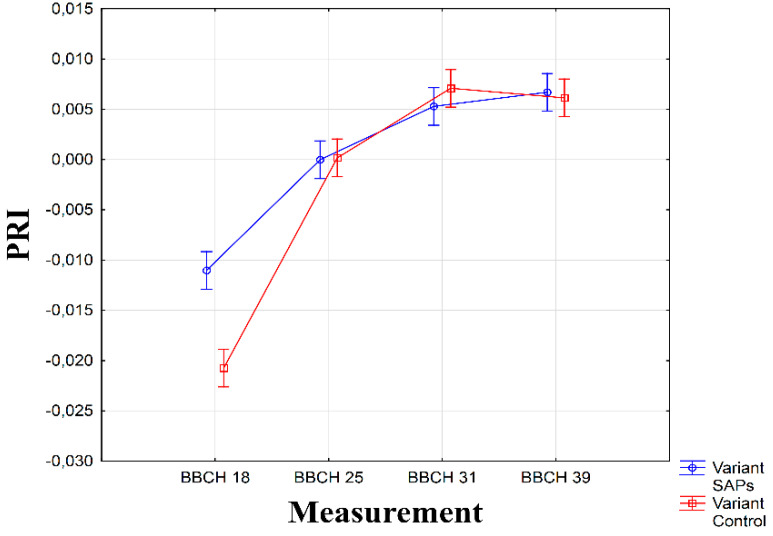
Interaction between experimental treatments and measurements for photochemical reflectance index (PRI). Figure was obtained at level of significance α = 0.01.

As can be seen from Figure 5, a marked deviation of PRI values was recorded in the first measurement with SAPs values significantly higher (α ≤ 0.01). In the remaining measurements, no significant differences were recorded (α > 0.05). Therefore, it was shown that the application of SAPs to seed can positively influence the level of photosynthesis intensity in dry conditions, especially at the beginning of the vegetation period (up to BBCH 19), and therefore can be used as a reliable indicator of drought.

**Figure 6:**
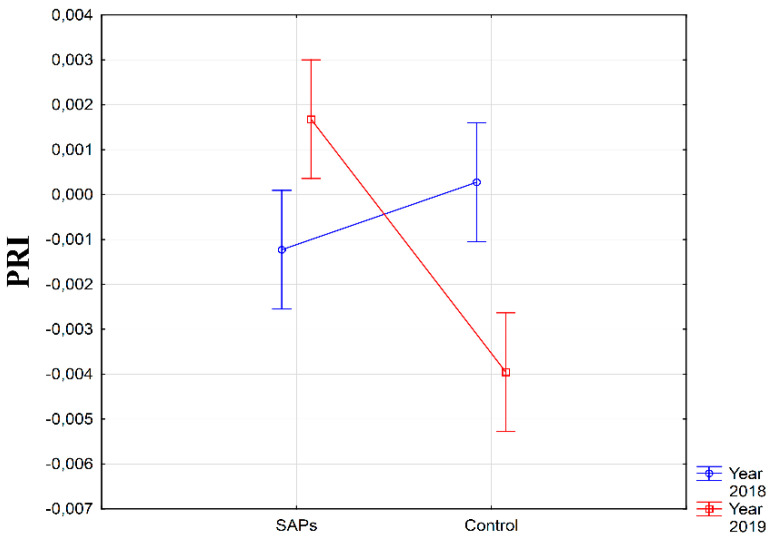
Interaction between experimental treatments and years for photochemical reflectance index (PRI). Figure was obtained at level of significance α = 0.01.

Considerable differences in PRI values were recorded in the comparison of the experimental years on the monitored experimental conditions. In the year 2019, the average recorded PRI value in the SAPs condition was significantly higher than the value in the control condition. In 2018, the average value of PRI was higher in the control condition; however, the difference in comparison with the SAPs condition was not statistically significant (Figure 6). This effect was expected as the course of weather conditions in each year was markedly different.

**Figure 7:**
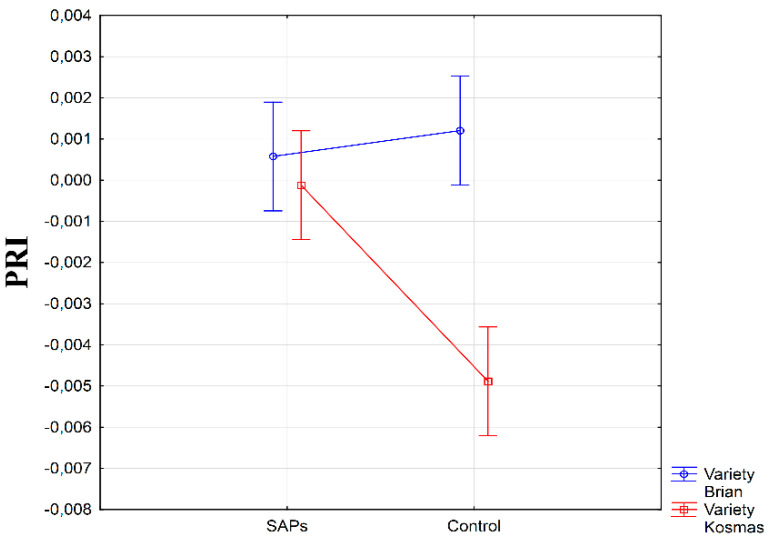
Interaction between experimental treatments and varieties for photochemical reflectance index (PRI). Figure was obtained at level of significance α = 0.01.

When comparing the test varieties, higher values of PRI were measured on the variety Brian; however, they were slightly elevated in the control condition when compared to the SAPs condition (α > 0.05). In contrast, the Kosmas variety attained lower average values of PRI, but with a statistically significant difference (α ≤ 0.01) between the conditions with the SAPs values higher (Figure 7). It is possible to assume that these substantial differences were caused by different genetic bases of the used varieties, but the adaptation ability of the Kosmas variety after SAPs application is noteworthy.

### 2.3. Evaluation of Production Parameters

**Figure 8:**
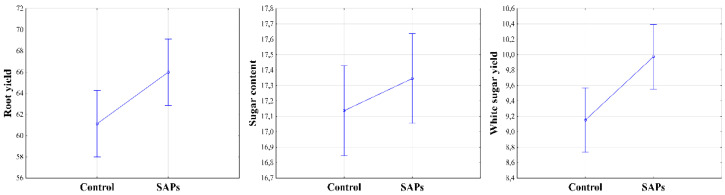
Root yield (t ha^−1^), sugar content (%) and white sugar yield (t ha^−1^) depending on experimental treatments. Figures with RY and WSY were obtained at level of significance α = 0.01, figure with SC was obtained at level of significance α = 0.05.

As listed in Table 1, there was a statistically significant impact of condition on the final root yield (RY) and on white sugar yield (WSY). Significant differences between SAPs and control conditions were confirmed statistically. The condition with SAPs application had an increase in root yield of 4.85 t ha^−1^ (rel. 7.35%, α ≤ 0.01) and in white sugar yield of 0.82 t ha^−1^ (rel. 8.22%, α ≤ 0.01) (Figure 8). This result was expected given the course of the experiment and the observed physiological parameters, although the white sugar yield is dependent not only on root yield but also on qualitative parameters of the roots. In quality evaluation, it was found that the sugar content (SC) was not influenced by the different conditions, which was also supported by further analysis, and the difference between the SAPs and control conditions was not statistically significant (α > 0.05).

### 2.4. Relationships between Physiological and Production Parameters

**Figure 9:**
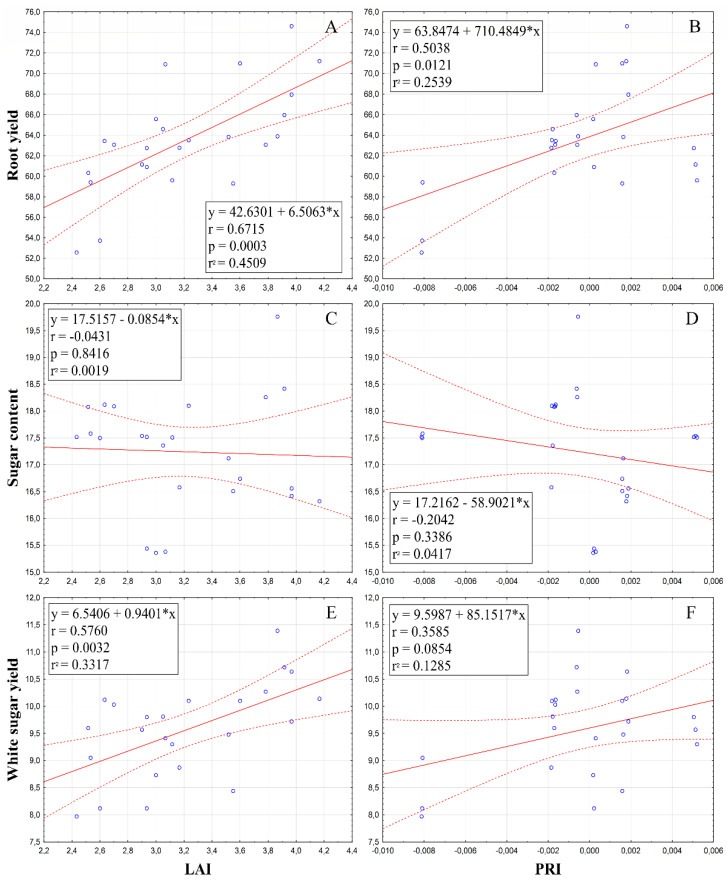
Relationships between leaf area index (LAI), resp. photochemical reflectance index (PRI) and production parameters: root yield (**A**,**B**); sugar content (**C**,**D**); white sugar yield (**E**,**F**). Linear equations, correlation coefficient, probability and regression are inserted inside graphs.

As can be seen in Figure 9A,C,E, the values of LAI were in a significant strong positively correlated relationship with the value of root yield (r = 0.6715**) and in a moderate positively correlated relationship with the white sugar yield parameter (r = 0.5760**), whereas the correlation between LAI and sugar content was not found (r = −0.0431). This supports our hypothesis that if we can positively influence the leaf area index by applying SAPs, it will result in a higher yield of the crop.

Similarly to LAI, photochemical reflectance index (PRI) was in a positive significant correlation with the root yield (*r* = 0.5038*), and a weak positive relationship with white sugar yield (*r* = 0.3585) was found. However, a weak negative correlation was found for PRI and sugar content (*r* = −0.2042) (Figure 9B,D,F).

Based on these results, it can be concluded that higher values of physiological parameters (LAI, PRI) have positive impact on the increase in quantitative parameters (RY and WSY) of sugar beets, but qualitative characteristics (SC) were not significantly impacted.

**Figure 10:**
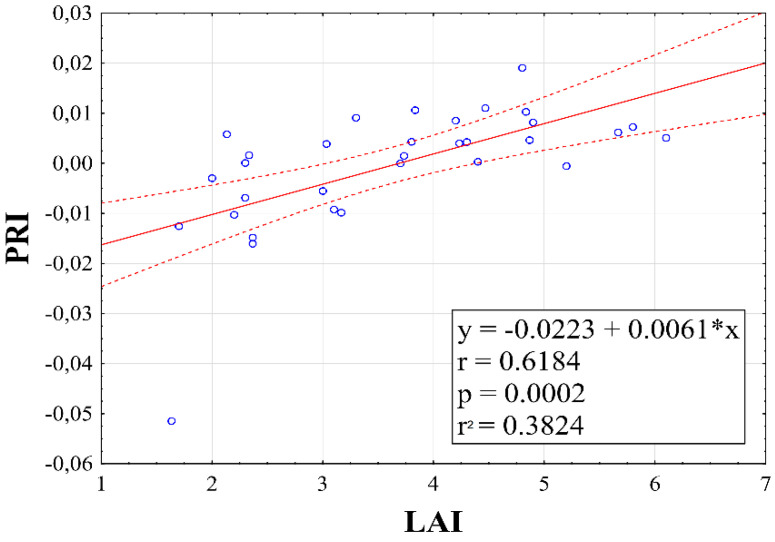
Relationship between leaf area index (LAI) and photochemical reflectance index (PRI). Linear equations, correlation coefficient, probability and regression are inserted inside graphs.

As can be seen from Figure 10, a mutual correlation analysis of PRI and LAI parameters confirmed a statistically significant correlation (*r* = 0.6184**). This was expected as the growth of assimilation apparatus of a plant is closely related to the photosynthesis process.

## 3. Discussion

The impact of drought on the production of field crops, including sugar beets, has been examined in many studies [52,53,54]. The importance of this limiting factor has been increasing especially in recent years in connection with ongoing climate change [55,56]. Several parts of Europe are also affected, where owing to missing or uneven rainfall during vegetation, the crops suffer water shortage resulting in yield decrease. Sugar-beet water requirements are varied and, depending on the variety, range from 100 to 600 mm per year [57]. This was confirmed in our research as the rainfall in 2018 and 2019 ranged 197.4—354.2 mm and the reactions of the varieties to the given amounts were different. In the monitored years, the distribution and not the amount of the rainfall can be considered the biggest water-management problem. Although the amount of the rainfall ranged in the middle values of the crop requirement, a large proportion of it was in the form of storm rainfall followed by a long period of drought. The decrease of yield due to drought is a complex system, which includes the intensity of the drought and its length, but also the phenological phase of the plant during the drought [58,59]. Our experiment was focused on the quantification of the drought impact in the early phases of sugar-beet growth using spectral indexes (LAI and PRI) and on the possibilities of mitigating its impact using superabsorbents applied to seed in the context of achieved yield and quality. To a large extent, drought limits the growth and absorption of CO_2_ by plants, and the use of leaf area index is suitable to evaluate this reaction [60]. In [61], it was found that drought and its impact on plant growth and development can be determined using LAI. Another index that can be used to evaluate the reaction of the plant to changing agroecological conditions is PRI. PRI is a spectral index, which can be used as an indicator of photosynthesis intensity [62] using two near reflectance bandwidths (531 nm and 570 nm) closely related to the carotenoid pigment cycle [63]. The results obtained in this experiment show that superabsorbents have a positive impact on mitigating the drought consequences at the beginning of vegetation, which was demonstrated by higher values of PRI and LAI parameters compared with the control condition. By measuring PRI during vegetation, a significant impact of SAPs on the intensity level of photosynthesis of sugar-beet canopy was found. The application of these substances to the seed can positively influence the productivity of the canopy. Monitoring of drought and its influence on field crops using spectral indexes has received much attention worldwide [35,64,65]. One way to achieve higher yield values is the use of suitable genetic material for the specific cultivation area. In this study, we found different reactions of varieties to environmental conditions, which was demonstrated in the results of PRI and LAI evaluations. This is consistent with [10], which states that canopy closure is different among varieties. Multiyear research on sugar beets was conducted to determine the positive correlations of spectral indexes (LAI and PRI) with yield parameters (RY, WSY, SC). The influencing of photosynthetic, biochemical, and other internal processes using different seed treatment materials (e.g., superabsorbents) to increase the yield and quality remains a challenge in the research community. In this study, we found strong correlations between spectral indexes (LAI and PRI) and the values of quantitative parameters (RY and WSY), and we believe that the increased values of the spectral indexes in the SAPs condition had a significant effect on the final yield of the sugar beets. However, the authors in [66] found that the formation of the leaf apparatus and the corresponding sugar-beet yield have no correlation relationship. Similar investigations have been conducted in the past by several researchers [67,68,69].

## 4. Materials and Methods

### 4.1. Characteristics of Location and Soil Conditions

**Figure 11:**
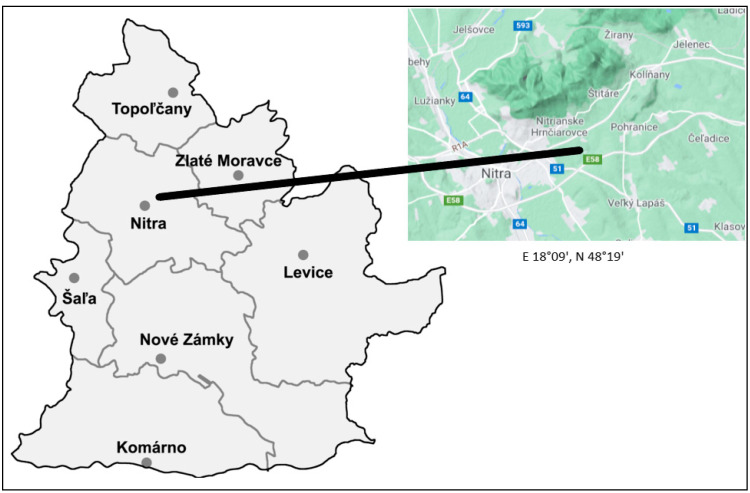
Illustration of experimental locality. Area is located in Podunajská nížina—Lowland, near Nitra city and Zobor hill in southwestern part of Slovak Republic.

The experiment was started in 2018 and 2019 on the research fields of Slovak University of Agriculture in Nitra. They are situated in the Danubian Lowland (E 18°09′, N 48°19′) with flat relief (Figure 11). From the perspective of climatic characteristics, it is a dry and very warm area, with an average rainfall of 539 mm and average temperature of 10.2 °C. The fields contain medium heavy loamy soil with medium-low humus content (± 2.20% depending on the year and pre-crop) and weakly acidic soil reaction (pH 5.50).

The pre-crop for the sugar beet in both years was common wheat (*Triticum aestivum* L.). After the pre-crop harvest, the after-harvest remains were plowed. Prior to soil tillage, soil samples were collected (0.3 m) for laboratory analysis of the macronutrient content, soil pH, and humus content. The content of inorganic nitrogen was determined using the calorimetric method, the ammonia form of nitrogen using Nessler’s reagent [70], and nitrate nitrogen using phenol 2.4-disulfonic acid [71]. The phosphorus and potassium contents were determined using the Mehlich III. test [72]. The soil reaction was determined using a 1 molar KCl solution as described in [73]. The humus content in the soil sample was established based on Tjurin method [74]. Based on the found contents (Table 2), the dose of net nutrients was calculated using the nitrogen balance method [75]. Stable manure in the dose of 50 t ha^−1^ together with phosphorus and potassium were applied in the fall of each year.

### 4.2. Variety and Superabsorbent Characteristics

The experiment was conducted using the Kosmas and Brian varieties (Strube D&S GmbH, Söllingen, Germany) of sugar beets whose characteristics are suitable for growing in dry and warm climates. Kosmas is a genetically monogerm triploid variety of transitional normal-sugar-content type. Brian is a monogerm diploid hybrid with a high sugar content, tolerant to *Rhizomania* and *Cercospora beticola*.

The seed of the selected varieties was treated using the seed coating technology Aquaholder^®^Seed (PeWaS s.r.o., Bratislava, Slovak Republic). This technology is based on superabsorbent polymers, which are able to absorb up to 100 times their weight and subsequently release them to the roots during the dry period.

### 4.3. Experimental Design

The experiment was established with the randomized complete block design method [76]. Sowing was conducted using 12-row drill Monopill S (Kverneland group, Klepp, Norway) with precision-distance sowing of the sugar beets. The density of the crops was determined using row spacing of 0.45 m × 0.18 m. Each experimental condition was sowed in three replications. One experimental block was of 54 m^2^ area (10 m length × 5.4 m width). The date of the sowing was 18 April 2018 in the first year of the experiment and 2 April 2019 in the second year of the experiment.

### 4.4. Leaf Area Index Measurement

To objectively evaluate the growth of the sugar beet, especially in the initial vegetation phases, the leaf area index, which provides information on LAI and biomass production using photosynthetically active radiation (PAR), was chosen as the physiological parameter. Overall, LAI was measured six times (BBCH 18; 25; 31; 39; 46; 49) with SS1 SunScan Canopy Analysis System (Delta-T Devices Ltd., Cambridge, United Kingdom) as in [77,78].

### 4.5. Photochemical Reflectance Index Measurement

This measure evaluates the production abilities of the crops using the comparison of two wavelengths (531 nm and 570 nm), that is, whether they are in production or defense mode during the influence of various stressors. In [79], it is reported that PRI can be defined and calculated using the following formula:

PRI = (P_531_ − P_570_)/(P_531_ + P_570_); (1)

PRI was measured four times during the vegetation (BBCH 18; 25; 31; 39) using a nondestructive method with PlantPen PRI200 (Photon Systems Instruments Ltd., Brno, Czech Republic), similarly to [80,81]. The measurements were collected 10 times from adult leaves of five plants in each condition. For precision in measurement, strict measurement conditions were observed (cloudless sky, dry leaves, and measurement time).

### 4.6. Crop Harvest and Quality Analysis of the Sugar Beet

Two representative rows of sugar beets were selected in each experimental block, and harvest was performed by manual plowing out the roots. Subsequently, the sugar beets were weighed, and the obtained value was converted to yield per hectare. Then, homogeneous samples were sent for qualitative analysis of the sugar beet to a sugar production facility (Považský cukor a.s., Trenčianska Teplá, Slovak Republic), a member of the Nordzucker Group. The sugar content was determined using Venema auto-analyser IIIG (Venema Consulting, Groningen, Netherlands) as in [82].

### 4.7. Statistical Evaluation

The collected data were processed, analyzed, and graphically represented using Statistica 10 software (StatSoft, Inc., Tulsa, OK, USA). ANOVA was used to determine the influence of the factors on the monitored sugar-beet parameters. The individual conditions were subsequently tested using a Tukey test to determine significance at probability level (0.05 or 0.01).

Correlation analysis was used to determine the dependence between spectral indexes (LAI and PRI) and yield parameters (root yield, sugar content, and white sugar yield).

## 5. Conclusions

In this study, we focused on evaluating the impact of superabsorbent polymers on physiological and yield parameters of the sugar beet. The pre-sowing coating of seed in SAPs had a significant effect on the leaf area index, photosynthetic reflectance index, root yield, and white sugar yield, whereas an impact on sugar content was not found. In comparison with the control condition, it can be concluded that the most pronounced impact of SAPs was observed in the initial growth phases of the sugar beets, as demonstrated by the PRI and LAI parameters; however, the formation of biomass represented by LAI was higher throughout the duration of the experiment when using SAPs. In drought and heat stress conditions, the application of superabsorbents may increase the production of biomass or photosynthesis and thus attain sugar-beet yield potential. Furthermore, interactions of SAPs with other factors (year and variety) were investigated, and differences due to different weather courses and genetic bases were found. Correlation analysis confirmed a mutual positive correlation between physiological parameters as well as correlations of these parameters with root and white sugar yields. Finally, our results supported our hypothesis that the application of SAPs to seed significantly increases the drought resilience of plants, especially in the initial growth phases. Subsequently, this influenced the yield formation and the height of the final product, although without a statistically significant impact on root quality. The application of SAPs in plant production management is highly justified, especially in dry areas, which are constantly expanding.

## Figures and Tables

**Table 1:** Analysis of variance (ANOVA) for impact of different source of variation on production parameters: root yield (RY), sugar content (SC), white sugar yield (WSY) and physiological parameters: leaf area index (LAI), photochemical reflectance index (PRI).

Source of Variation	RY (t ha^−1^)	SC (%)	WSY (t ha^−1^)	LAI (m^2^ m^−2^)	PRI
	*p*-Values				
**Year**	0.3314	0.0000 **	0.0092 **	0.0036 **	0.1960
**Variety**	0.0138 *	0.0550	0.0612	0.0000 **	0.0000 **
**SAPs**	0.0061 **	0.2798	0.0011 **	0.0000 **	0.0000 **

* and **: significance at α ≤ 0.05 and at α ≤ 0.01, respectively.

**Table 2:** Nutrients and humus contents, soil pH in soil after analysis in autumn and spring, respectively.

Year	Nutrient Content mg kg^−1^			pH	Humus (%)
	N_an_	P	K		
2017/2018	25.18	93	385	6.28	1.72
2018/2019	10.00	63	315	6.69	1.60

## Data Availability

The data presented in this study are available upon request from the corresponding author.
